# Effects of SPAD value variations according to nitrogen application levels on rice yield and its components

**DOI:** 10.3389/fpls.2024.1437371

**Published:** 2024-10-21

**Authors:** Tae-Heon Kim, Suk-Man Kim

**Affiliations:** ^1^ Institute of Agricultural Science and Technology, Kyungpook National University, Daegu, Republic of Korea; ^2^ Department of Crop Science, Kyungpook National University, Sangju, Republic of Korea

**Keywords:** rice, SPAD value, low nitrogen conditions, yield components, grain yield

## Abstract

Nitrogen (N) is the most essential element for growth, development, and grain yield determination in crops. However, excessive nitrogen application can result in environmental pollution and greenhouse gas emissions that contribute to climate change. In this study, we used 158 rice genetic resources to evaluate the relationships between the soil and plant analysis development (SPAD) value and grain yield (GY) and its components. The SPAD value ranged between 30.5 and 55.8, with a mean of 41.7 ± 5.3, under normal nitrogen conditions (NN, 9 kg/10a), and between 27.5 and 52.3, with a mean of 38.6 ± 4.8, under low nitrogen conditions (LN, 4.5 kg/10a). Under NN conditions, the SPAD values were in the following order: *japonica* (43.5 ± 5.8), *Tongil*-type (41.7 ± 2.5), others (41.7 ± 5.2), and *indica* (38.3 ± 3.8). By contrast, under LN conditions, the SPAD values were in the following order: *Tongil*-type (40.4 ± 2.1), others (40.1 ± 4.5), *japonica* (39.6 ± 5.2), and *indica* (35.6 ± 3.9). The 158 genetic resources showed no correlation between SPAD and yield. Therefore, the low-decrease rate (LDR) and high-decrease rate (HDR) SPAD groups were selected to reanalyze the relationships between the surveyed traits. The SPAD values were positively correlated with 1000-grain weight (TGW) for both LDR and HDR groups (NN: 0.63, LN: 0.53), However, SPAD and GY were positively correlated only in the LDR group. For TGW, the coefficient of determination (*R* ^2^) was 20% and 13% under NN and LN conditions, respectively. For GY, *R* ^2^ values of 32% and 52% were observed under NN and LN conditions, respectively. Genetic resources with higher SPAD values in the LDR group exhibited the highest yield (NN: 1.19 kg/m^2^, LN: 1.04 kg/m^2^) under both NN and LN conditions. In conclusion, we selected 10 genetic resources that exhibited higher GY under both NN and LN conditions with minimal yield reductions. These genetic resources represent valuable breeding materials for nitrogen deficiency adaptation.

## 1 Introduction

Nitrogen is an essential macronutrient required for plant growth and development and is also crucial for synthesizing amino acids, proteins, nucleic acids, chlorophyll, and plant hormones (Fukushima and Kusano, 2014; Chen and Ma, 2015). A soil nitrogen deficiency leads to chlorosis in leaves, poor growth, and, finally, a decrease in crop productivity. Nitrogen is a key factor in sustainable crop production and played a crucial role in the increase in crop productivity during the Green Revolution. Growing rice, a staple for about half of the global population, requires substantial nitrogen fertilization to maximize grain yields (Good and Beatty, 2011). Modern rice varieties have been developed under normal or high nitrogen conditions to attain a short culm length (using the *sd1* gene), lodging resistance, and high grain yields (Han-Hua et al., 2006; Cho and Koh, 2007; Wei et al., 2011). However, only 30–40% of the applied nitrogen fertilizer is utilized by rice, with over 50% lost to microbial consumption or leaching (Cho and Koh, 2007; Robertson and Vitousek, 2009; Coskun et al., 2017). Therefore, the excessive application of nitrogen fertilizer does not help in increasing yield but decreases nitrogen use efficiency (NUE), leading to increased crop production costs and negative environmental impacts such as water pollution and the emission of greenhouse gases like methane (CH_4_) and nitrous oxide (N_2_O) (Fischer et al., 2010; Sun et al., 2016; Wang et al., 2016). The need for low-carbon agricultural practices has become increasingly prominent because of concerns regarding climate change. Thus, there is a growing demand for research on reducing nitrogen fertilizer applications (Xuejun and Fusuo, 2011; Kim et al., 2019; Rogelj et al., 2021). However, limiting nitrogen application can affect the plant’s growth and overall development, lowering grain productivity. Nitrogen is a major chlorophyll component and affects leaf greenness, growth, and crop productivity (Fageria et al., 2011). Nitrogen deficiency first manifests as changes in leaf color, linked to complex physiological processes such as photosynthesis metabolism and efficiency and the transfer of photosynthesis products. This hinders the identification of key traits related to this issue.

Various studies have reported the effects of nitrogen application levels on the chlorophyll and nitrogen contents in the leaves and the plant’s photosynthetic ability, which are related to crop productivity. As nitrogen application levels increase, the content of photosynthetic pigments, such as chlorophylls a and b and carotenoids, rises (Peng et al., 2021). The leaf chlorophyll content is closely related to photosynthetic ability and serves as a marker to evaluate the physiological status of the crop (Kalaji et al., 2016; Zhao et al., 2019). The soil and plant analysis development (SPAD) value can be measured using simple, rapid, and nondestructive methods for estimating the relative leaf chlorophyll content. The SPAD value is highly correlated with chlorophyll content in several crops including rice, maize, wheat, and soybean (Turner and Jund, 1991; Monje and Bugbee, 1992; Reeves et al., 1993; Zotarelli et al., 2003; Wakiyama, 2016). In particular, the chlorophyll content exhibits a positive relationship with photosynthetic ability in rice (Xu et al., 1997; Wang et al., 2023).

In this study, we used genetic resources to evaluate the SPAD value variations under low nitrogen conditions (i.e., conditions where nitrogen application was reduced by half) and analyzed how these changes correlated with grain yield and other traits. Moreover, we selected genetic resources that might ensure stable yields despite the reduced nitrogen application, suggesting these could help develop rice varieties adapted to low nitrogen conditions.

## 2 Materials and methods

### 2.1 Plant materials

One hundred fifty-eight rice genetic resources were used as the plant materials to determine the changes in the SPAD value, grain yield (GY), yield-related traits, and other agronomic traits under reduced nitrogen fertilizer application. The genetic resources comprised 78 *japonica*, 44 *indica*, 24 *Tongil*-types, and 12 others (four tropical *japonica*, four landraces, two aromatic varieties, and two unclassified).

### 2.2 Field trials and fertilization method

The field experiment was conducted over two years (2022 and 2023). The plant materials were sown in seedling trays (60 × 30 cm) on May 6^th^ of each year and transplanted on June 1^st^ with a spacing of 30 × 15 cm and one plant per hill in both normal nitrogen (NN) and low nitrogen (LN) plots. Each experiment was conducted in triplicate. The nitrogen application rates were 9.0 kg/10a for NN and 4.5 kg/10a for LN, with phosphorus and potassium at 4.5 kg/10a and 5.7 kg/10a, respectively. Nitrogen was applied in a basal: tillering: panicle proportion of 50:20:30, whereas phosphorus was applied entirely as basal, and potassium was split 50:30 (basal: panicle). All other rice cultivation practices followed the standard cultivation methods of rice (RDA, 2012).

### 2.3 Leaf color and major agronomic traits survey

The leaf color was measured using a SPAD 502 Plus (Konica Minolta, Japan) and expressed as SPAD values. Measurements were taken from the middle portion of the flag leaves 14 days after heading (DAH), with five plants per replication, totaling 15 plants per treatment (Wang et al., 2014, 2015). Major agronomic traits such as days to heading (DTH), culm length (CL), panicle length (PL), and number of panicles per plant (NPP) were surveyed according to the standard evaluation system (RDA, 2012) with three plants per replication.

For statistical analysis, genetic resources were categorized into two groups based on the decrease in SPAD values under different nitrogen fertilization conditions: low-decrease rate (LDR) and high-decrease rate (HDR). The LDR group comprised 13% (20 out of 158) of the genetic resources and showed a slight decrease in SPAD values. By contrast, the HDR group (also 13%) showed the most substantial decrease in SPAD values.

### 2.4 Evaluation of grain yield and yield-related traits

Yield-related traits such as 1000-grain weight (TGW), number of spikelets per panicle (NSP), percentage of ripened grains (PRG), and GY were evaluated. Three plants per treatment were harvested at maturity and TGW was calculated by averaging the weight of 200 filled grains, measured in triplicate. NSP was determined by adding the number of filled and unfilled grains of a plant and dividing them by the number of panicles. PRG was determined as the percentage of filled grains out of the total number of seeds per plant. GY was calculated by multiplying the number of plants per square meter (28) by the average seed weight per plant, which was obtained by weighing the seeds from three plants per replicate. All yield-related trait evaluations were conducted according to the standard evaluation system (RDA, 2012).

### 2.5 Statistical analysis

Statistical analysis was performed using R version 4.2.2 software. T-tests, correlation, regression, ANOVA, and Scheffé tests were used to compare means and variances within genetic resources and among treatments. The corrplot, agricolae, ggplot2, and ggpubr R packages were used for correlation, regression, ANOVA, and Scheffé tests, respectively. Data used for regression analysis was normalized using the bcPower function from the car R package.

## 3 Results

### 3.1 Evaluation of SPAD values based on nitrogen fertilization levels

**Figure 1:**
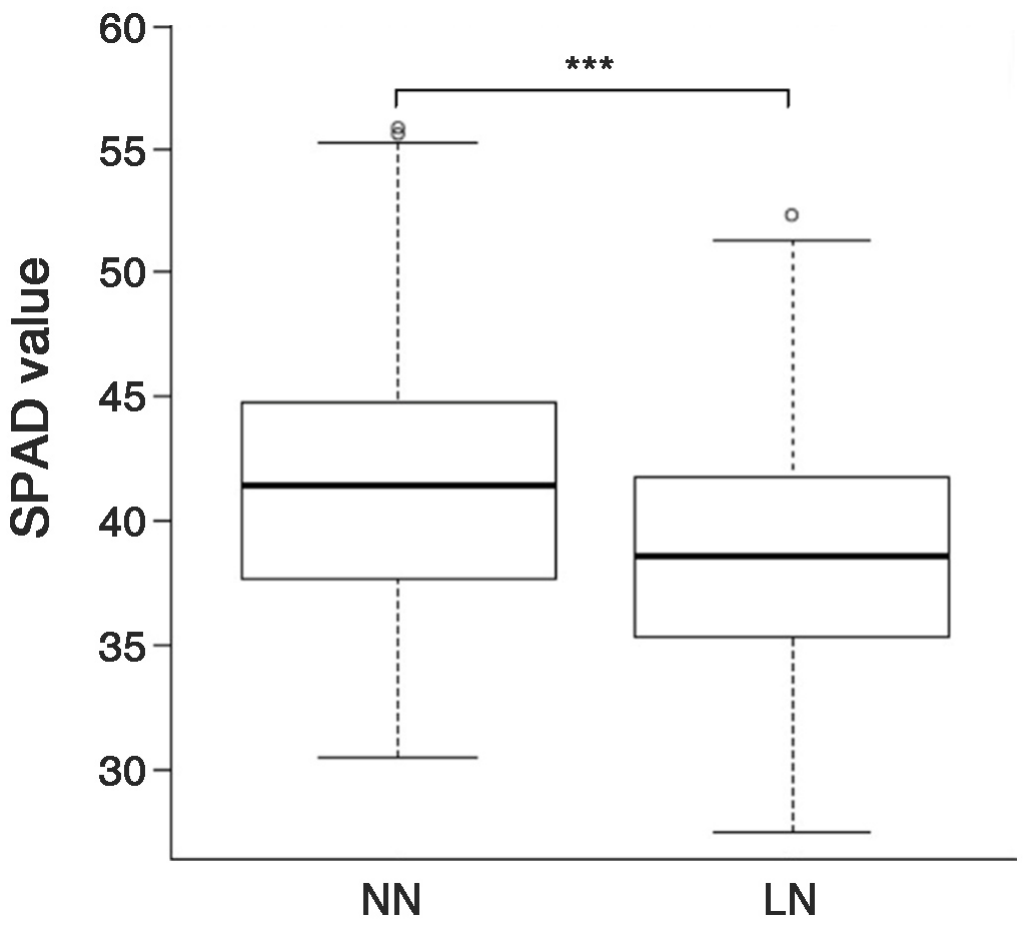
SPAD values of the rice genetic resources grown under two nitrogen application levels. NN, normal nitrogen conditions (9 kg/10a), LN, low nitrogen conditions (4.5 kg/10a). *** Indicates significant differences at *p* < 0.001 by a t-test.

The SPAD values of 158 genetic resources were evaluated based on nitrogen fertilization levels. As a result, the average SPAD value was 41.7 ± 5.3 (ranging from 30.5 to 55.8) under normal nitrogen (NN) conditions. By contrast, the average value decreased to 38.6 ± 4.8 (ranging from 27.5 to 52.3) under low nitrogen (LN) conditions (**Figure 1**). The SPAD values of the genetic resources significantly decreased by 7.3% under LN conditions compared to NN conditions.

**Figure 2:**
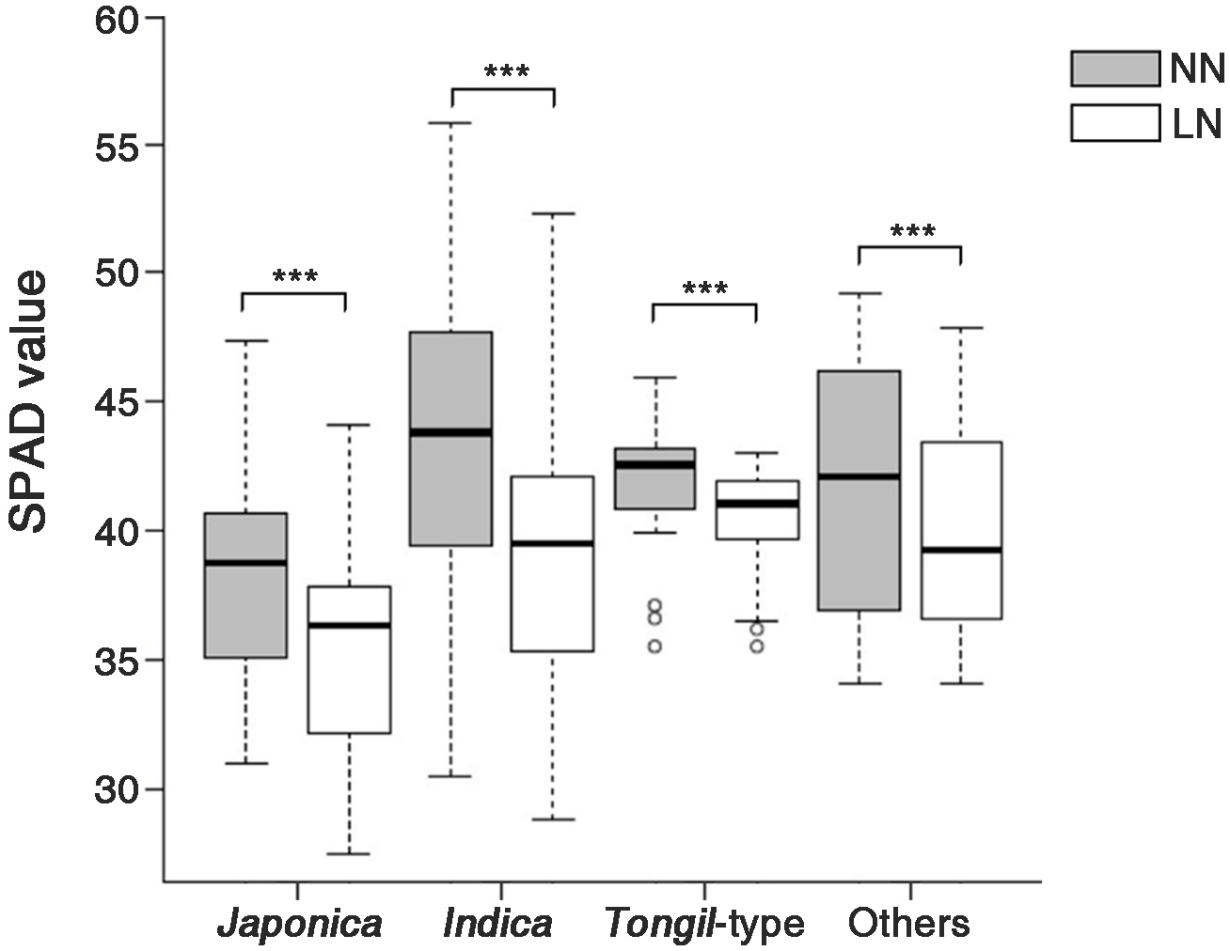
SPAD values of the rice genetic resources, divided into four groups according to ecotype, grown under two nitrogen application levels. NN, normal nitrogen conditions (9 kg/10a), LN, low nitrogen conditions (4.5 kg/10a). *** Indicates significant differences at *p* < 0.001 by a t-test.

Moreover, differences in the SPAD values were observed in the genetic resources categorized into four groups by ecospecies and ecotype (**Figure 2**). Under NN conditions, *japonica* showed the highest SPAD value (43.5 ± 5.8), whereas *indica* showed the lowest value (38.3 ± 3.8). The SPAD values under NN conditions were ranked as follows: *japonica* (43.5 ± 5.8) > *Tongil*-type (41.7 ± 2.5) > other (41.7 ± 5.2) > *indica* (38.3 ± 3.8). By contrast, under LN conditions, the rankings were: *Tongil*-type (40.4 ± 2.1) > other (40.1 ± 4.5) > *japonica* (39.6 ± 5.2) > *indica* (35.6 ± 3.9). Compared to NN conditions, a significant decrease in SPAD values ranging from 3.1% to 9.1% was observed across all ecotypes under LN conditions. *Japonica* exhibited the greatest decrease in SPAD values (9.1%), whereas *Tongil*-type showed the lowest rate of reduction at 3.1%.

### 3.2 Correlations among SPAD values, yield, and yield components

A correlation analysis was conducted to analyze the effects of the SPAD value variations on the major agricultural traits of the tested genetic resources under NN and LN conditions (**Supplementary Figure S2**). Under NN conditions, the SPAD values were negatively correlated with the yield components NPP (-0.45) and PRG (-0.36), whereas TGW was the only positively correlated trait (0.49). Although the correlation coefficients for the investigated traits slightly varied under LN conditions compared to NN conditions, the correlation patterns were similar.

### 3.3 Correlations for the investigated traits in groups divided according to their decrease in SPAD values

**Figure 3:**
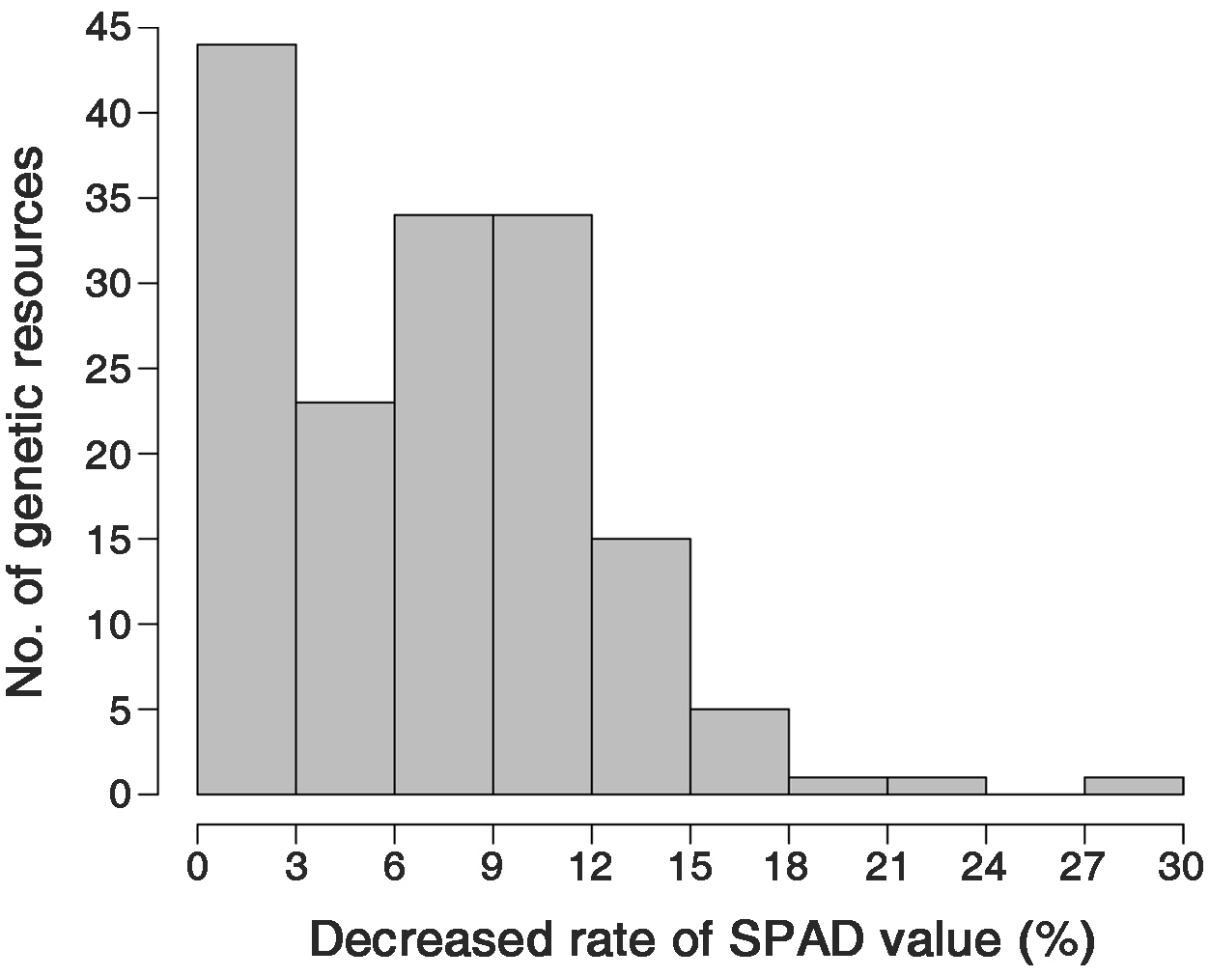
Frequency distribution of the percentage decrease in the SPAD value of the low nitrogen (LN) conditions compared to the normal nitrogen (NN) conditions.

The decrease in the SPAD values observed as the conditions changed from NN to LN ranged from 0.0% to 28.7%, resulting in a nearly normal distribution (**Figure 3**). This suggests that SPAD value variations due to nitrogen fertilization levels are quantitative traits determined by multiple genes. Initially, no correlation was found between the SPAD values and GY across all genetic resources (**Supplementary Figure S2**). Therefore, genetic resources were divided into LDR and HDR groups based on their decrease in SPAD values under LN conditions. The HDR group exhibited a decrease in SPAD value ranging from 12.5% to 21.1%, while the LDR group showed a decrease of 0.0% to 1.3% (Data not shown).

**Figure 4:**
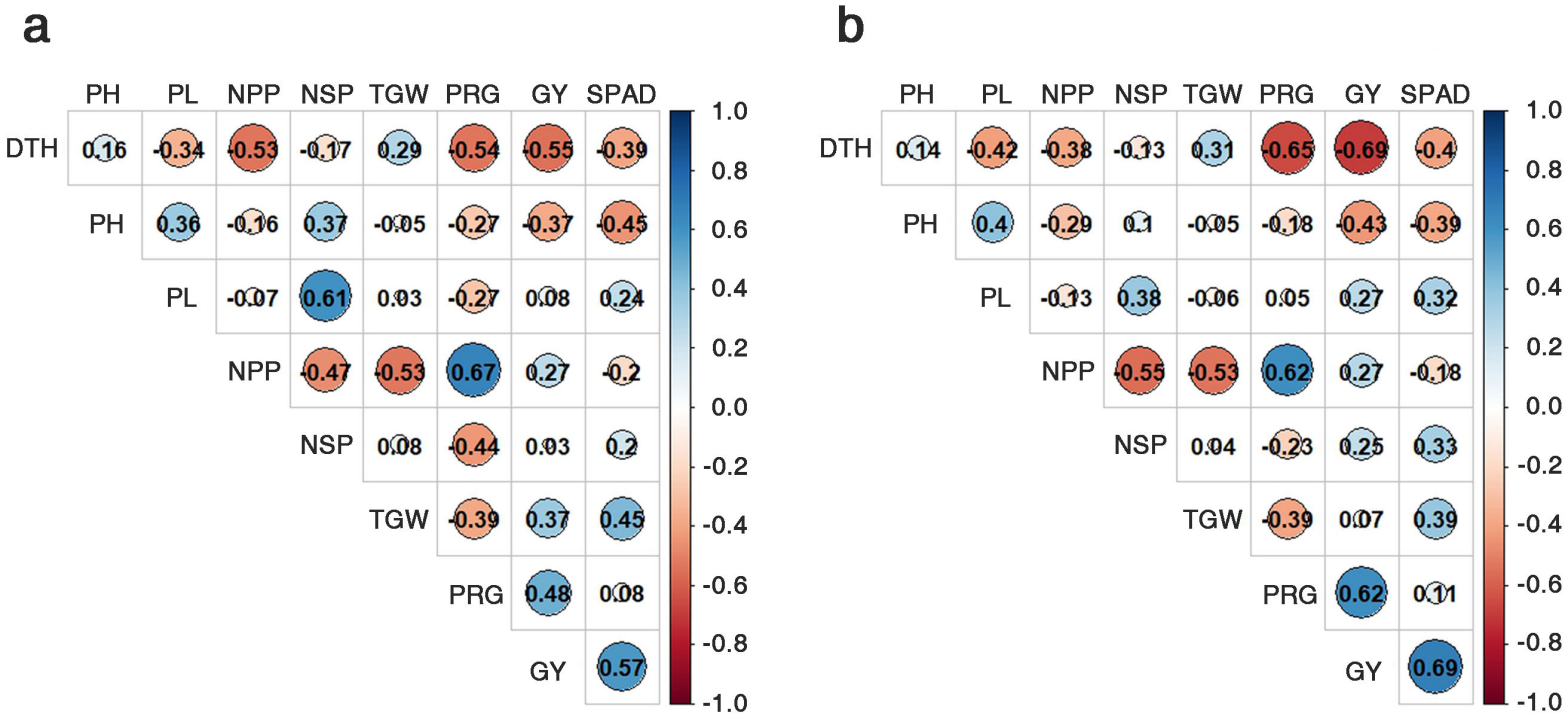
Correlation co-efficiency among nine traits, using the 20 genetic resources of the LDR group. Normal nitrogen conditions **(A)** and low nitrogen conditions **(B)**. DTH, days to heading; CL, culm length; PL, panicle length; NPP, no. of panicles per plant; NSP, no. of spikelets per panicle; TGW, 1000-grain weight; PRG, percentage of ripened grains; GY, grain yield.

This facilitated the correlation analysis between the SPAD values and GY. The correlation analysis within the LDR group revealed that both nitrogen fertilizer levels (NN and LN) were positively correlated with TGW (NN: 0.45, LN: 0.39) and GY (NN: 0.57, LN: 0.69), while negatively correlated with the NPP (NN: -0.20, LN: -0.187) (**Figure 4**). For the HDR group, only the SPAD values and TGW were positively correlated for both nitrogen levels (NN: 0.63, LN: 0.53), with other traits being negatively correlated or not correlated (**Supplementary Figure S3**). In summary, the SPAD values were positively -correlated with TGW in both the HDR and LDR groups but positively correlated with GY only in the LDR group (**Figure 4**; **Supplementary Figure S3**).

### 3.4 Regression analysis between SPAD values and yield-related traits across nitrogen fertilization levels

**Figure 5:**
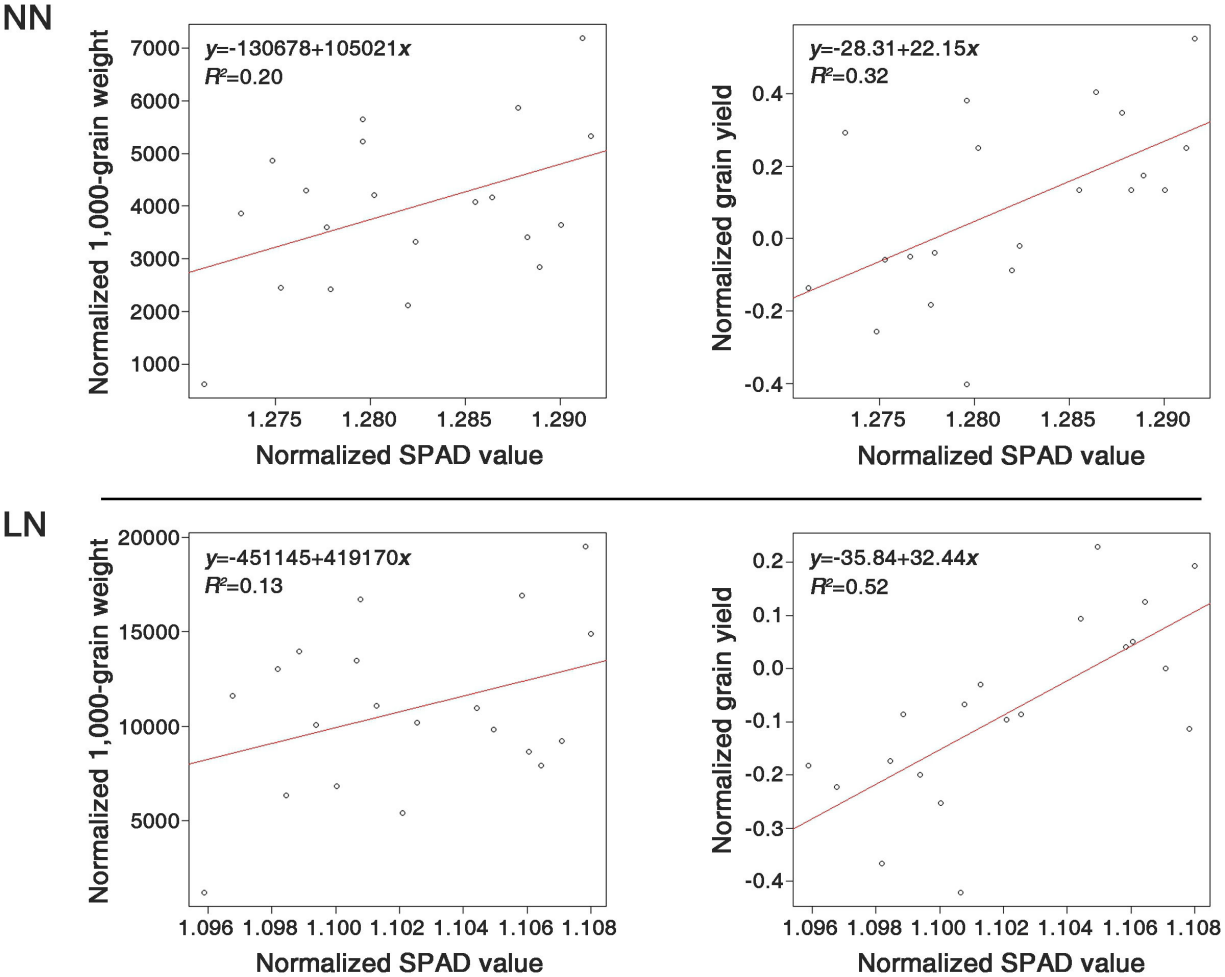
Comparison of the determination coefficients between SPAD values and two traits, 1000-grain weight (TGW) and grain yield (GY), under normal nitrogen (NN) and low nitrogen (LN) conditions, using the 20 genetic resources of the LDR group. All the compared components were normalized by the bcPower function of the car package in R (version 4.2.2).

A regression analysis was conducted within the LDR group to evaluate the correlations between the SPAD values and the investigated traits. The SPAD values served as the independent variable, while TGW and grain GY were the dependent variables (**Figure 5**). For the LDR group, the coefficients of determination were 20% and 13% for TGW under NN and LN conditions, respectively, whereas for GY, they were 32% and 52%, respectively.

### 3.5 Comparison of individual GY within selected groups

**Figure 6:**
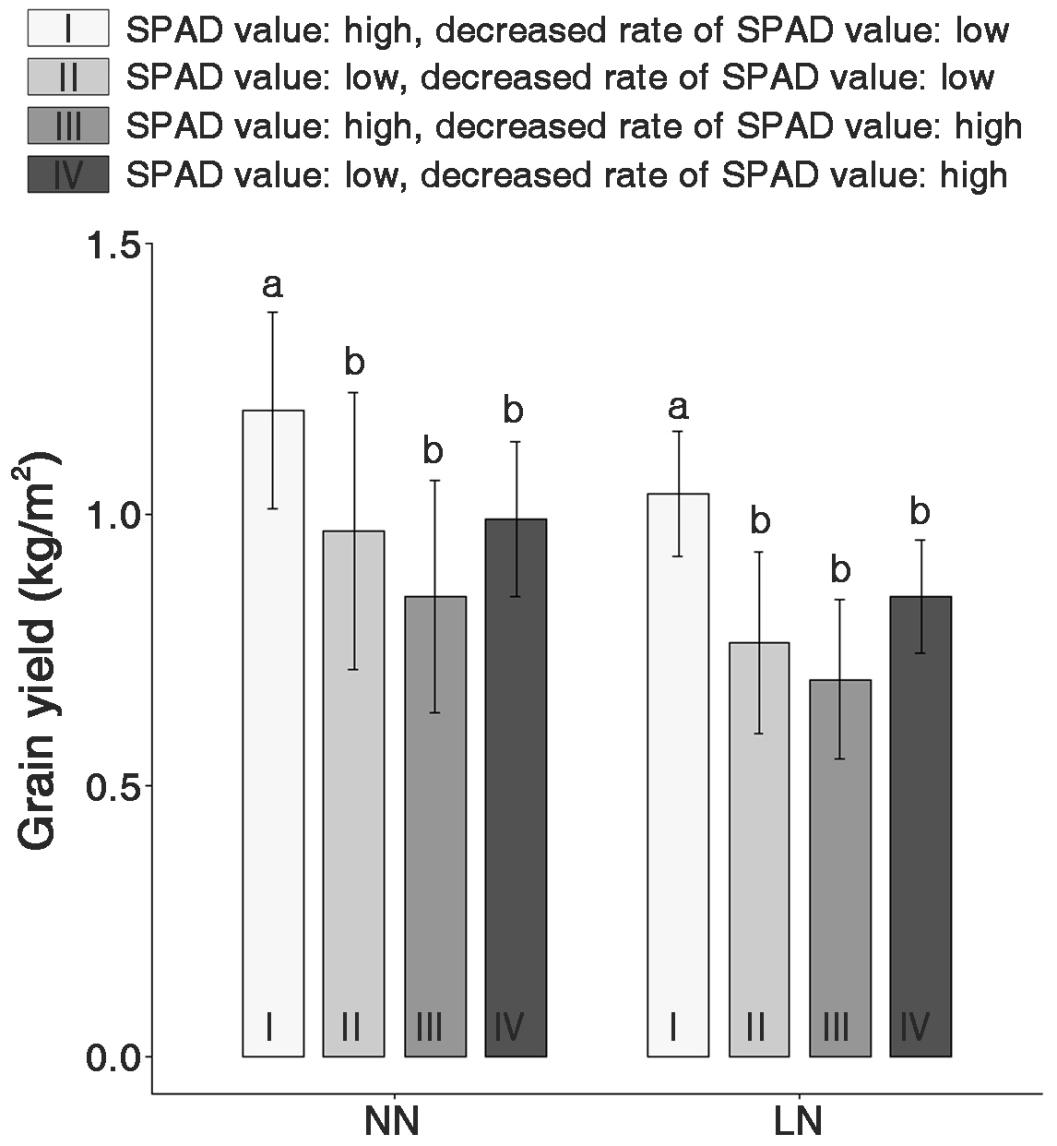
Comparison of the grain yield (GY) in four groups classified based on the SPAD values and decreased SPAD values under normal nitrogen (NN) and low nitrogen (LN) conditions. I and II are groups with higher and lower SPAD values in the LDR group, respectively. III and IV are groups with higher SPAD and lower SPAD values in the HDR group, respectively. The lowercase letters ‘a’ and ‘b’ in each nitrogen condition indicate significant differences based on one-way ANOVA tests (p < 0.01, Scheffé test).

Based on SPAD value variations due to nitrogen fertilization levels, genetic resources in the LDR and HDR groups were divided into four subgroups to evaluate the GY (**Figure 6**). Under NN conditions, Group I (with high SPAD values), an LDR subgroup, exhibited the highest GY, with no significant differences among the other groups. This trend was consistent even under low nitrogen conditions. Moreover, a noticeable decrease in GY ranging from 14.6% to 21.1% was observed across all groups under LN conditions. Yield decrease due to low nitrogen application was observed across all genetic resources.

### 3.6 Selection of nitrogen deficiency adaptive resources

**Figure 7:**
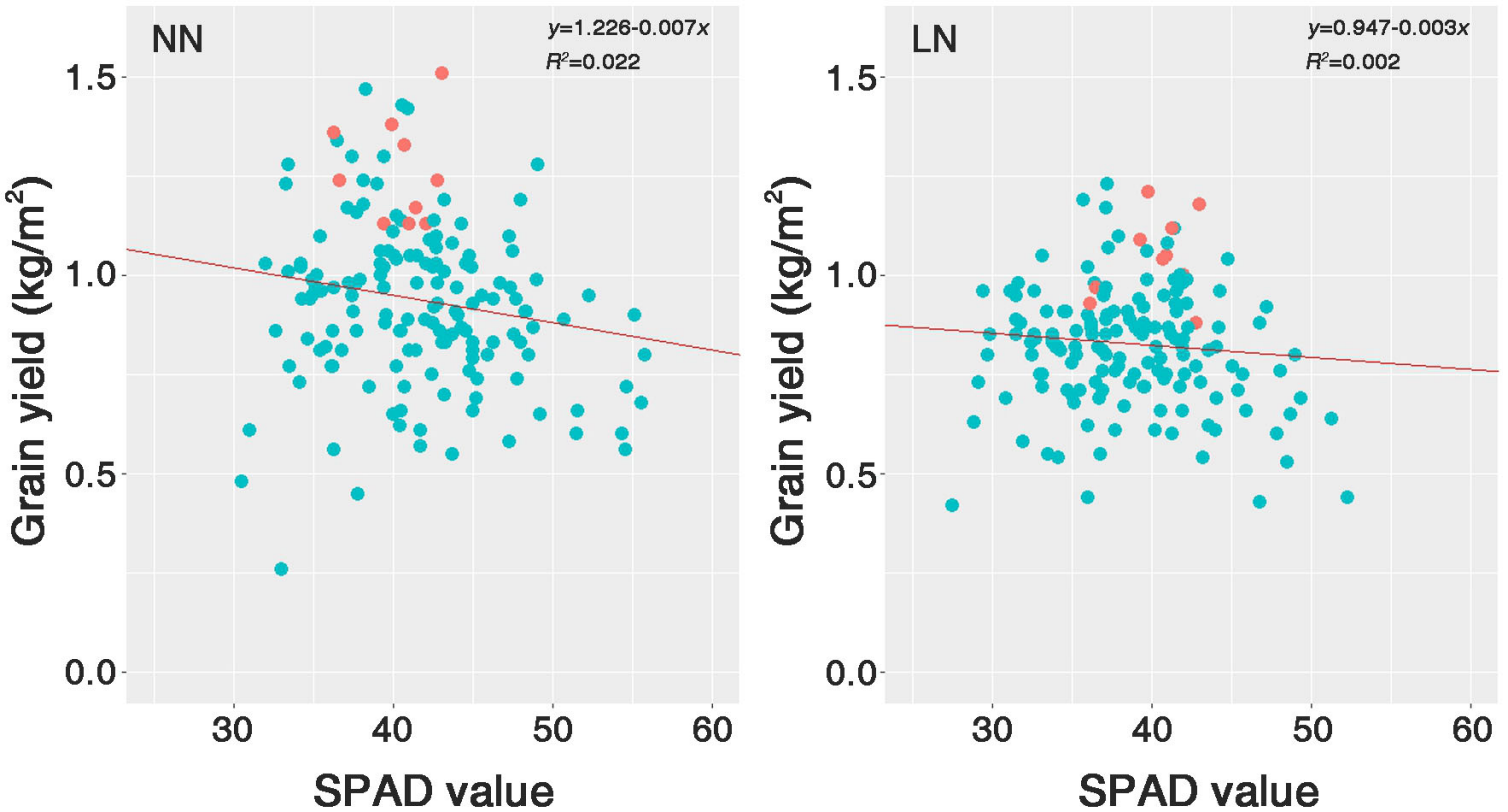
Scatter plots of the SPAD value against the grain yield (GY) for 158 rice genetic resources grown under two nitrogen conditions. The red dots indicate the positions of the 10 selected genetic resources within Group I, an LDR subgroup. NN, normal nitrogen conditions (9 kg/10a), LN, low nitrogen conditions (4.5 kg/10a).

Based on the statistical analysis, we selected 10 resources that exhibited the lowest decrease in GY under LN conditions (**Supplementary Table S1**). The selected genetic resources comprise six *Tongil*-type, three *indica*, and one *japonica*. These genetic resources were located at the top of the SPAD value distribution and GY scatter under both nitrogen fertilization conditions (**Figure 7**). *Japonica* represented the largest proportion of the total genetic resources (49.4%). However, according to the SPAD values, it was underrepresented among the selected nitrogen deficiency adaptive resources because it was the most sensitive to nitrogen fertilization levels.

## 4 Discussion

Reducing nitrogen inputs in agriculture is part of the efforts to reduce carbon emissions to address climate change. Rice requires large nitrogen fertilizer inputs throughout its lifespan to ensure productivity (Zhu and Chen, 2002). Therefore, efforts are urgently needed to improve the nitrogen use efficiency of crops under low nitrogen conditions. We evaluated the GY, major agronomic traits, and SPAD values to explore the feasibility of predicting grain yields based on chlorophyll content estimation in rice plants grown under low nitrogen conditions.

*Japonica* has higher leaf greenness than *indica* and its growth and grain yield are more affected by low nitrogen conditions (Oka, 1958; Ichii and Tsumura, 1989). We found that *japonica* had the highest SPAD value among the tested plant materials (43.5 ± 5.8), whereas *indica* had the lowest (38.3 ± 3.8) (**Figure 2**; **Supplementary Figure S1**). The SPAD value decreased most significantly in the *japonica* ecotype (by 9.1%) under LN conditions. The decrease in leaf greenness is a phenotypic variation observed with lower nitrogen amounts and is associated with decreased photosynthetic ability (Xiong et al., 2015; Zhao et al., 2019).

The SPAD value was positively related to TGW under both NN (0.49) and LN (0.44) conditions (**Supplementary Figure S2**). As this value increases, so does the leaf nitrogen content (Wang et al., 2014). The increase in the leaf nitrogen content enhances the plants’ photosynthetic ability (Zhao et al., 2019). An increase in the SPAD values enhances the content of the rubisco enzyme, the gross photosynthetic rate, and the maximum quantum yield of PSII (Fv/Fm), all of which are associated with photosynthetic ability in rice (Huang and Peng, 2004; Kato et al., 2004; Kumagai et al., 2009). The correlations observed among the investigated traits suggest that the chlorophyll content is associated with carbohydrate (i.e., starch and soluble sugars) accumulation in developing seeds.

The decrease in the SPAD values as conditions changed from NN to LN showed a nearly normal distribution (**Figure 3**). This suggests that SPAD value variations due to nitrogen fertilization levels are quantitative traits determined by multiple genes. Although we found no correlation between the SPAD values and GY across all genetic resources (**Supplementary Figure S2**), the SPAD value variations under different nitrogen fertilization levels may interact with factors determining yield or yield performance. Thus, a correlation analysis between SPAD values and GY is challenging. Hence, we conducted an additional statistical analysis on the investigated traits of the genetic resources according to nitrogen fertilization levels, controlling for the key factor (SPAD values). An analysis of SPAD values and decrease rates across the 158 genetic resources according to rice ecotypes revealed that *indica* exhibited the highest SPAD values among the ecotypes, along with the greatest decrease rates (**Figure 2**; **Supplementary Figure S1**). Meanwhile, in the HDR and LDR groups, the decrease rate of SPAD values was not specific to any particular ecotype. The LDR group consisted of 25% *indica*, 25% *japonica*, 35% *Tongil*-type, and 15% others. The HDR group consisted of 25% *indica* and 75% *japonica* (Data not shown). Thus, while SPAD values and their decrease rates may exhibit certain tendencies based on ecotype, they are not strictly determined by the ecotype of the rice.

Under NN and LN conditions, the SPAD values were positively correlated with TGW in both the HDR and LDR groups but they were positively correlated with GY only in the LDR group (**Figure 4**; **Supplementary Figure S3**). This result suggests that the gene responsible for increasing the chlorophyll content and the gene that ensures chlorophyll’s stability under LN conditions exhibit additive effects. This additive effect is observed in the LDR group but not in the HRD group, leading to increased GY at high SPAD values in the group possessing both genes. TGW was positively correlated with GY in the LDR group under both NN and LN conditions. Therefore, higher SPAD values enhanced TGW, which, as a yield component, benefits GY, underlining the importance of chlorophyll content in crop productivity.

In this study, when nitrogen application rates were reduced, 85% (34 out of 40 genetic resources) of the genetic resources in the top and bottom 13% for TGW showed little change in their rankings (Data not shown). This suggests that TGW is a yield component that is most stable against environmental variations and can be partially increased by enhancing SPAD value (chlorophyll content). The stability of TGW under environmental variations and the potential for TGW to be increased by SPAD value observed in this experiment represent different characteristics of TGW.

Regression analyses were conducted between the SPAD values and GY in the LDR group, resulting in determination coefficients of 32% and 52% under NN and LN conditions, respectively (**Figure 5**). This result indicates that even with variations in nitrogen application rates, genetic resources with consistent SPAD values can significantly predict grain yields. The fact that 52% of the variance in GY is explained by the SPAD values under LN conditions suggests the potential for developing new phenotypic markers. Furthermore, identifying genes associated with NUE could help enhance nitrogen efficiency in rice varieties through marker-assisted breeding. The 10 rice genetic resources selected in this study exhibit characteristics that minimize yield reduction under LN conditions (**Supplementary Table S1**). These genetic resources can be used to develop rice varieties adapted to low nitrogen conditions.

## Data Availability

The raw data supporting the conclusions of this article will be made available by the authors, without undue reservation.
