# Supplementary material for: Effects of SPAD value variations according to nitrogen application levels on rice yield and its components
Source: Front Plant Sci. 2024 Oct 21;15:1437371. doi: 10.3389/fpls.2024.1437371 (PMC11533161; doi:10.3389/fpls.2024.1437371)
Supplement: Supplementary file 1 [file Image1.pdf]

frontiers

Supplementary Material

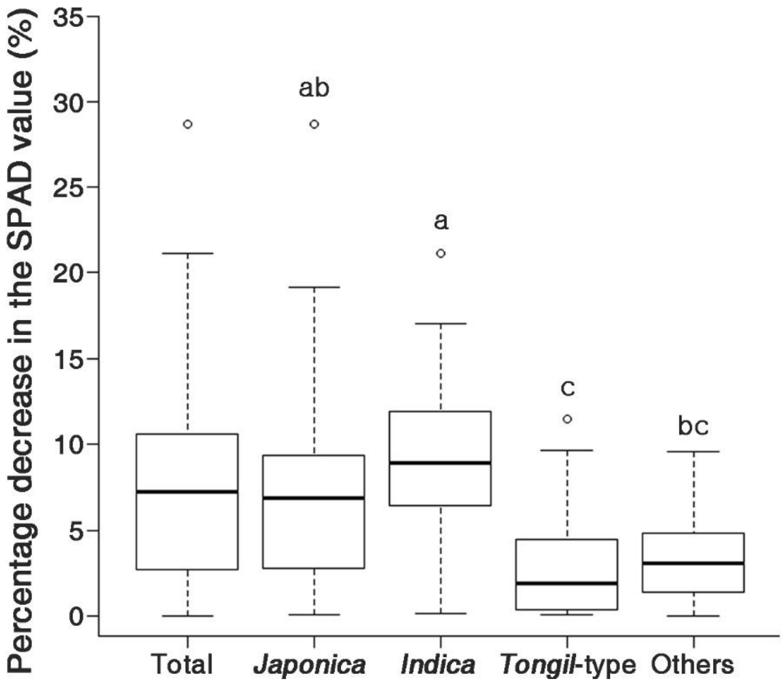

Supplementary Figure S1. Percentage decrease in the SPAD values for 158 rice genetic resources grown under two nitrogen fertilization conditions. Lowercase letters within four ecotypes (*Indica*, *Japonica*, *Tongil*-type, and others) refer to one-way ANOVA tests ($p < 0.01$, Scheffé test).

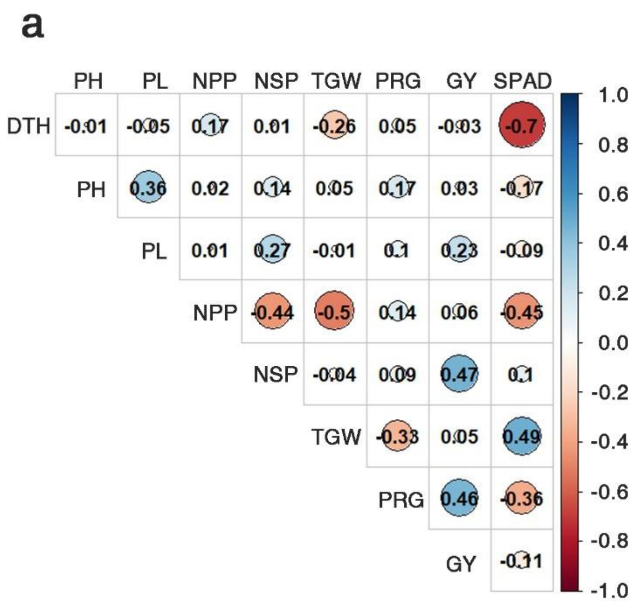

Supplementary Figure S2. Correlation coefficients of nine traits in 158 rice genetic resources. Normal nitrogen conditions (a) and low nitrogen conditions (b). DTH: days to heading, CL: culm length, PL: panicle length, NPP: no. of panicles per plant, NSP: no. of spikelets per panicle, TGW: 1000-grain weight, PRG: percentage of ripened grains, GY: grain yield.

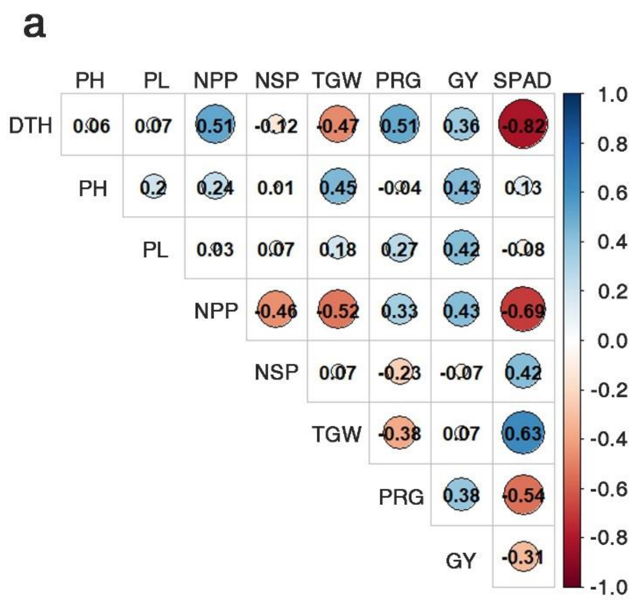

Supplementary Figure S3. Correlation coefficients of nine traits in the 20 genetic resources of the HDR group. Normal nitrogen conditions (a) and low nitrogen conditions (b). DTH: days to heading, CL: culm length, PL: panicle length, NPP: no. of panicles per plant, NSP: no. of spikelets per panicle, TGW: 1000-grain weight, PRG: percentage of ripened grains, GY: grain yield.
